# Financial burden of tuberculosis diagnosis and treatment for patients in Ethiopia: a systematic review and meta-analysis

**DOI:** 10.1186/s12889-024-17713-9

**Published:** 2024-01-22

**Authors:** Dawit Getachew Assefa, Zewdu Gashu Dememew, Eden Dagnachew Zeleke, Tsegahun Manyazewal, Ahmed Bedru

**Affiliations:** 1 KNCV Tuberculosis Foundation, Addis Ababa, Ethiopia; 2 Management Science for Health (MSH), Addis Ababa, Ethiopia; 3 Ohio State Global One Health Initiative, Addis Ababa, Ethiopia; 4 College of Health Sciences, Center for Innovative Drug Development and Therapeutic Trials for Africa (CDT-Africa), Addis Ababa University, Addis Ababa, Ethiopia

**Keywords:** Tuberculosis, Cost, Catastrophic, Ddiagnosis, Treatment, Ethiopia

## Abstract

**Background:**

Despite the diagnosis and treatment of tuberculosis (TB) given free of charge in many high-burden countries, the costs that patients face in the cascade of care remain a major concern. Here, we aimed to investigate the financial burden of TB diagnosis and treatment for people with TB in Ethiopia.

**Method:**

For this systematic review and meta-analysis, we searched PubMed/MEDLINE, Embase, and Cochrane Center for Clinical Trials from December 1 2022 to 31 June 2023 for articles reporting the cost of diagnosis and treatment for patients regardless of their age with all forms of TB in Ethiopia. Major study outcomes were catastrophic costs, direct (out-of-pocket) pre-diagnosis, medical cost, and post-diagnosis costs, indirect (income loss) costs, coping costs, and total costs. We have used a threshold of 20% to define catastrophic costs. We used random-effects meta-analyses to calculate summary estimates of costs. R-studio software was used for analysis. The study is registered with PROSPERO: CRD42023387687.

**Result:**

Twelve studies, with a total of 4792 patients with TB, were included in our analysis. At the 20% threshold of total expenses, 51% of patients (2301 participants from 5 studies, 95% CI: 36-65%, I^2^ = 97%) faced catastrophic costs due to bacteriologically confirmed drug-sensitive pulmonary TB. Private facility diagnosis, drug-resistance TB, TB-HIV co-infection, hospitalization, and occupation were found to be associated with catastrophic costs. Reduction in the total cost spent by the patients was associated with digital adherence interventions, community-based direct observed therapy, short-course MDR-TB treatment regimens, and active case-finding. Pre-diagnosis costs had a positive correlation with diagnosis delays and the number of facilities visited until diagnosis. Post-diagnosis costs had a positive correlation with rural residence and inpatient treatments.

**Conclusion:**

Irrespective of a national policy of free TB service, more than half of TB patients are suffering catastrophic costs due to drug-sensitive pulmonary TB in Ethiopia and most of the patients spend a lot of money during the pre-diagnosis period and intensive phase, but declined drastically over time. Active case-finding, digital adherence interventions, community-based treatment, and comprehensive health insurance coverage have the potential to minimize the financial burden of TB diagnosis and treatment.

## Introduction

Tuberculosis (TB) infection is one of the top ten causes of death, with more than one million deaths worldwide in 2019 [1]. According to the 2023 World Health Organization (WHO) report, the majority of TB cases in 2022 occurred in the Southeast Asia region (accounting for 46%), followed by Africa (23%) and the Western Pacific (18%) [2]. Ethiopia is one of the 30 high TB burden countries with an estimated TB incidence of 126/100,000 [2].

Tuberculosis imposes significant financial burdens due to factors such as its prolonged treatment, diagnostic procedures, and the use of multiple drugs. Hospitalization, which can lead to reduced productivity, is also a potential cost factor [3, 4]. In a global survey of 27 countries, the incidence of catastrophic total costs (defined as sum of direct costs and indirect costs divided by annual household income, as estimated from self-reported income, greater than 20%) for TB patients and their households ranged from 13% (95% CI: 10–17%) in El Salvador to 92% (95% CI: 86–97%) in the Solomon Islands [5]. Even with a 10% threshold, half (50%) of households affected by TB experienced catastrophic health expenditures [6]. Costs incurred by a TB patient include either direct or indirect costs. The direct costs comprise out-of-pocket expenses for medical and nonmedical services whereas the indirect costs constitute lost income because of lost workdays [7]. These costs can also drive families into poverty. An important goal of the End-TB strategy is there should not be families affected by TB-related catastrophic costs by 2020 [8].

Even with TB diagnosis services offered for free in public healthcare facilities and treatment services available in both public and private healthcare facilities, a recent systematic review highlighted a significant financial strain on patients, particularly those dealing with MDR-TB [9]. A study indicated that 70.6% of the total cost comprised indirect costs, with 29.4% being direct costs [4]. An extended cost-effectiveness analysis for expanded TB control in Ethiopia demonstrated that active case finding could reduce TB-related deaths by 27% and catastrophic costs by 32%. Enhancing DOTs for DS-TB was projected to prevent 25% of deaths and 15% of catastrophic costs, while improvements in MDR-TB care were estimated to avert up to 1% of deaths and 6% of catastrophic costs in Ethiopia from 2018 to 2035 [10].

Therefore, understanding the catastrophic cost of TB would seem a relevant issue for policy and decision-making. To our knowledge, there have been no systematic reviews and meta-analyses of the financial burden and catastrophic health expenditure of TB in Ethiopia. In this study, we aimed to investigate the financial burden of TB diagnosis and treatment for patients in Ethiopia.

## Methods

This protocol has been registered at the International Prospective Register of Systematic Reviews (PROSPERO) database, ID: CRD42023387687 [11]. The methods and findings of the review were reported according to the preferred reporting items for systematic reviews and meta-analyses (PRISMA 2020) [12].

### Eligibility criteria

The PICOS format has been used to identify eligible studies.

### Participants

Patients who are affected by all forms of TB, regardless their age and gender, were included.

### Outcomes

To estimate patients’ costs, we have used the tool developed by TB CAP (KNCV Tuberculosis Foundation, the WHO Global TB Program, and the Japan Anti-Tuberculosis Association) in consultation with many other organizations and individuals who have been working on TB patients’ costs [13].

### Primary outcome

1. Catastrophic cost is the sum of direct costs and indirect costs divided by annual household income, as estimated from self-reported income, greater than 20%.

### Secondary outcomes

2. Direct out-of-pocket patient expenses during the pre-diagnosis period (incurred from onset of illness to treatment initiation) were determined by asking patient expenses at each visit for consultation, laboratory tests, drugs, transportation, meals, and lodging.
3. Direct costs consisted of out-of-pocket charges for medical services (consultation, drugs, laboratory tests, X-ray, and hospitalization) and nonmedical services (transportation, meal, and accommodation) while visiting healthcare facilities.
4. Post-diagnosis direct costs (incurred from initiation to completion of the prescribed treatment) were measured by inquiring about patients’ medical and nonmedical expenses during visits for anti-TB treatment.
5. The indirect costs were estimated using the human capital approach. Patients were requested to estimate the time lost due to sickness and visits for consultation, hospitalization, drug collection, and trip journey.
6. Total cost was both pre- and post-diagnosis direct out-of-pocket expenses (for medical and nonmedical services), indirect costs were measured, and cost of TB including pain and suffering (willingness to pay).

### Studies

Studies that assessed the cost of diagnosis and treatment of TB in Ethiopia have been included.

### Exclusion criteria

Studies conducted in various countries that did not provide separate results for Ethiopia were excluded.

### Electronic searches

A systematic literature search has been done to identify relevant articles from online databases PubMed/ MEDLINE, Embase, and Cochrane Center for Clinical Trial database (CENTRAL). The search was done according to guidance provided in the Cochrane Handbook for Systematic Reviews of Interventions from December 1, 2022 to June 31, 2023. The search terms were tuberculosis [MeSH Terms]) OR (Myco-bacterium tuberculosis[MeSH Terms])) OR (Koch’s disease[MeSH Terms])) AND (catastrophic cost[MeSH Terms])) OR (total cost[MeSH Terms])) OR (out of pocket cost[MeSH Terms])) OR (direct cost[MeSH Terms])) OR (indirect cost[MeSH Terms])) OR (TB-related out of pocket expenditure[MeSH Terms])) OR (TB-related catastrophic health expenditure[MeSH Terms])) OR (Catastrophic cost of TB/HIV co-infection[MeSH Terms])) OR (Catastrophic cost of MDR TB patients[MeSH Terms])) OR (TB related catastrophic costs[MeSH Terms])) OR (Catastrophic cost TB patients[MeSH Terms])) AND (Ethiopia[MeSH Terms]).

### Study selection, data collection, and data analysis

The Cochrane Handbook for Systematic Reviews of Interventions has been followed. Furthermore, R-studio software has been used.

### Selection of studies

**Fig. 1:**
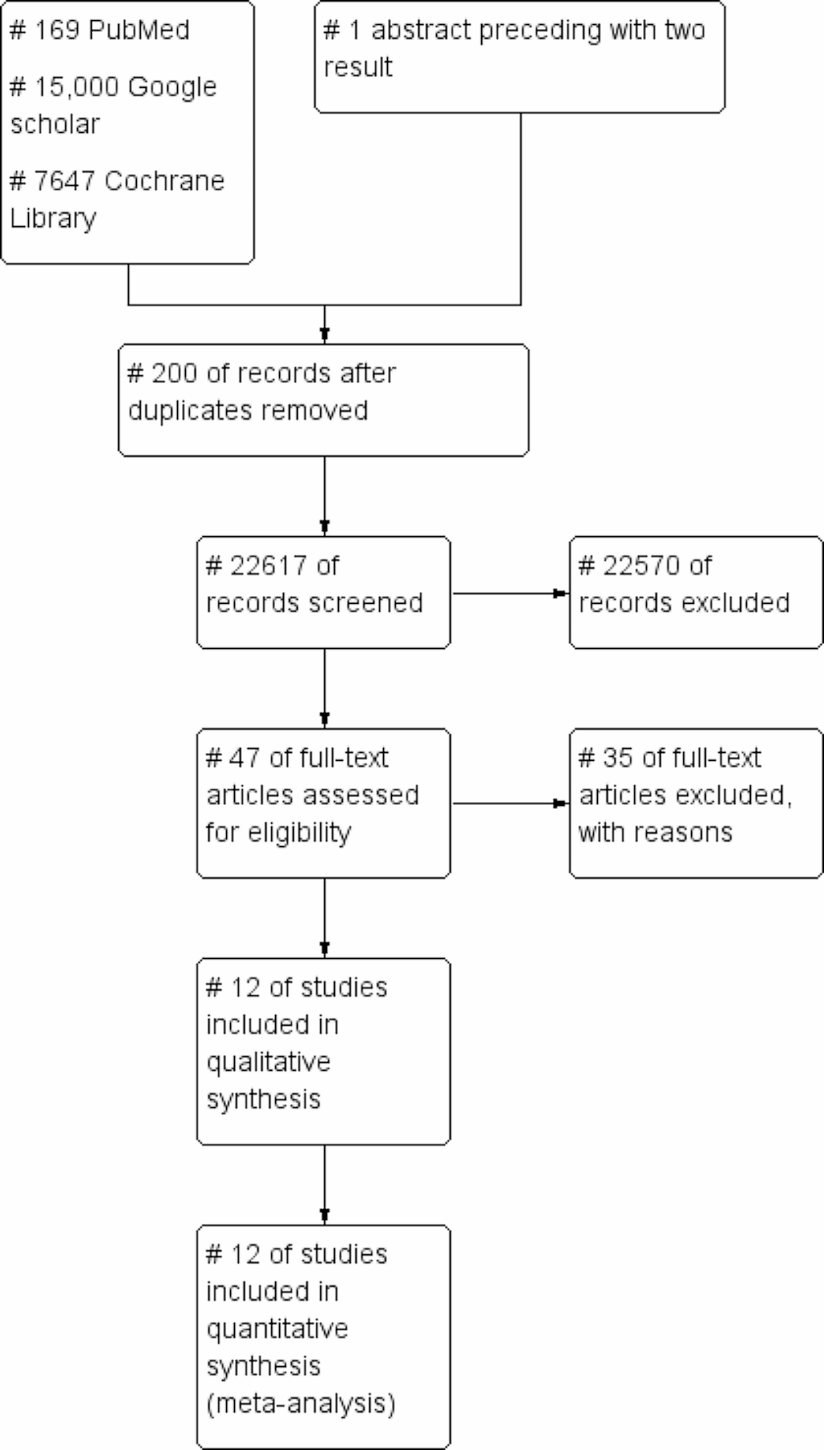
PRISMA flow diagram

To import the research articles from the electronic databases and remove duplicates, we have used ENDNOTE software version X7. The searched literature and full-text copies of all potentially related articles have been independently reviewed by two authors (DGA and EDZ). Also, multiple publications from the same dataset have been checked and studies were included in this review based on the inclusion criteria. Any disagreements have been resolved through discussion. The PRISMA flow chart has been used to report the screening and selection process Fig. 1.

### Data extraction and management

The title and abstract produced from the electronic search were also independently screened by two reviewers (DGA and EDZ). Information gathered from the studies included details on methods, participants, interventions, and outcomes. Additionally, relevant data such as title, journal, year of publication, study design, study setting, type of TB infection, study period, sample size, baseline characteristics of study subjects, direct and indirect costs, and catastrophic costs were extracted from each article using a structured extraction format presented in tabular form. Any discrepancies between the reviewers have been settled by consensus. Two author (DGA and EDZ) independently extracted data and information collected was cross-checked by another investigator (AB).

### Assessment of risk of bias in included studies

The methodological quality for cross-sectional studies was assessed using the Newcastle-Ottawa quality assessment scale and the risk of bias for each trial was evaluated by two authors (DGA and EDZ) independently using the Cochrane Collaboration’s tool for assessing the ‘Risk of bias’ [14]. The risks were classified as high risk, unclear risk, and low risk.

### Measures of effect size

Risk ratio (RR) escorted by 95% CIs has been used to report pooled results of dichotomous outcomes.

### Assessment of heterogeneity

Heterogeneity between the included articles was evaluated by looking at the forest plots (to detect overlapping CI) and the Cochrane Q and I^2^ statistic has been used to quantify heterogeneity among the included studies in each analysis, the Chi^2^ test with a *P* < 0.10 used to suggest statistical significance, and the results have been elucidated following Cochrane Handbook for Systematic Reviews of Interventions Version 6.0, Chap. 10: Analyzing data and undertaking meta-analyses.

- 0–40%: might not be important;
- 30–60%: may show moderate heterogeneity;
- 50–90%: may show substantial heterogeneity;
- 75–100%: considerable heterogeneity.

### Data synthesis

The meta-analyses were done coherently with the Cochrane recommendations. To help reading, individual codes have been given to include studies together with the first author, and year of publication. Since the studies were conducted by different researchers and managed independently, the random effect model was used.

## Result

A total of 22,817 studies from databases were searched, of which 47 full-text studies for eligibility were assessed; 12 fulfilled the inclusion criteria for meta-analysis and for qualitative analysis Fig. 1.

### Characteristics of the included studies

Twelve studies enrolled 4792 participants were included in this review. The majority of the studies were cross-sectional and three of them were randomized control trials Table 1.

### Methodological quality and risk of bias

The risk of bias for assessments in cross-sectional studies is summarized in Table 2. The risk of bias assessment for randomized controlled trials showed that six domains were low risk for bias in one study [15] and each three domains were also low risk for bias in two studies [16, 17]. The rest of the domains was either high risk or unclear for bias. The majority of the studies employed a tool created by the Stop-TB partnership for cost estimation [4, 7, 18–20], two studies [16, 21] tilized the WHO TB patient cost surveys and methods for the economic evaluation of healthcare programs [3, 22]. In two studies, the tools used were not specified [23, 24] and one study employed thresholds of 10%, 20%, and 40% to define catastrophic costs [25].

**Table 1:** Characteristics of the included studies

Author, Year	Study design	Population criteria study duration	Study setting	Sample size and age	Results	Predictors
Abayot, 2018(4)	Longitudinal study	New cases, older than 18 years, and on intensive phase treatment.	10 districts of southwestern Ethiopia.	735 new cases Age- median (IQR) of 27(20–37) years.	-**Total pre-diagnosis cost**- median (IQR) cost of US$97.6 (56.4–184.2) and direct cost constitutes 25.6%. -**Total post-diagnosis cost**-median (IQR) of US$93.75 (56.9–141.54) and direct cost constitutes 35.9%. - **Total cost**- median (IQR) of US$201.48 (136.70–318.94) and total direct cost constituted 29.4%. - For 471/569 (82.8%) of the cases, the total cost represents more than 10% of their estimated household annual income.	-**For Pre- diagnosis cost**- patient and provider delays, being clinically diagnosed, TB diagnosis at private facilities, and the number of visited healthcare facilities. **-For post- Dx cost**- being a rural resident, having a travel time beyond 1 h to the treatment center, being admitted for anti-TB treatment, patient, and provider delays. - **Total cost**- rural residence, travel time to treatment center beyond 1 h, action taken before HCF visit, hospitalized for anti-TB treatment, number of visited HCF, and patient and provider delays.
**Abraha, 2023** (26)	Cross-sectional study	TB patients	Oromia, Amhara, Southwest Ethiopia People, South Nation and Nationalities and Peoples Region, and Sidama.	432 and 397 TB patients	-Catastrophic cost at 20% threshold in 2020 66% and in 2022 43.8%. - Baseline and follow-up mean direct and indirect costs were 2.17US$ and 1.62US$, respectively. - Direct medical costs, direct non-medical costs, and indirect costs at baseline were 11.6%, 76.2%, and 12.3%. -Direct medical costs, direct non-medical costs, and indirect costs during follow-up were 30.4%, 19.2%, and 52.4%.	
**Belete, 2010**(27)	Cross-sectional study	Adult TB patients	Public health facilities in Addis Ababa.	− 604 TB patients. - Age- NA	**-**Total costs of TB illness to patients during DOT mean (SD) $177.3 (78.7) and the direct (OOP) cost mean (SD) $123.0 (58.8). **-**Direct mean treatment follow-up costs were $23.5. **-**OOP payments were catastrophic for 63% of TB patients. **-**Only 56 (9.7%) of TB patients had any kind of medical insurance scheme. -For the majority (90%) of TB patients, OOP payments were covered by their family members.	**- The total cost**- patient’s household income, residence, need for additional food, and primary income.
**Collins, 2018** (18)	Cross-sectional study	MDR-TB patients.	The University of Gonder Hospital, Saint Peter’s TB Specialized Hospital, and All Africa Leprosy, Tuberculosis and Rehabilitation Training Centre (ALERT) Hospital.	169 MDR-TB patients. - Age- NA	- Total cost-US$1378. - Pre-diagnosis cost- US$83. - Intensive phase- US$661 and continuous phase US$634. - The impact on the patient’s employment and overall patient and family income was generally catastrophic: 74% of all respondents reported losing their jobs, 66% of patients lost household income, and household income was reduced by 38%.	NA
**Daniel, 2010** (16)	Community-randomized trial	Smear-positive patients.	Southern Nations, Nationalities, and Peoples’ Regional State	**-**229 smear-positive patients. **-**Age- mean (SD) 26.8 (13.7) for community DOT and 25.2 (11.8) years for health facility DOT.	**-** The cost of anti-TB drugs for a patient was US$22.1. - The transport and food costs were US$0.9 and US$2.8, respectively. -The cost per successfully treated patient was US$161.9 in health facility DOT and US$60.7 in community DOT.	NA
**Jason, 2020** (17)	Phase-III randomized controlled trial.	MDR-TB patients.	St. Peter’s Specialized Hospital and the Armauer Hansen Research Institute Hospital.	**-119** MDR-TB patients. - Age- NA	- The cost was greater with the long than for the short regimen: the total cost per participant was US$ 6096.6 versus US$ 4552.3. - The mean cost of a serious adverse event in Ethiopia was higher for the long (US$ 82.1; 95% CI: 46.0 to 118.2) than the short regimen (US$ 15.7; 95% CI: 1.2 to 30.2). -The total direct costs per participant were US$ 575.4 for the long regimen and US$ 337.3 for the short regimen.	NA
**Lelisa, 2020** (25)	Cross-sectional survey.	Individuals seeking TB care.	27 health facilities in Afar and Oromia regions.	− 787 individuals seeking TB care. -Age- mean (SD) 30 (14) years.	**-** The mean (SD) patient cost of HIV was $115 ($118) per TB episode. - Total direct cost of TB constituted 46%. - The total mean (SD) indirect cost was $63 ($83) per TB episode. -The productivity loss related to TB follow- up visits accounts for 36% of the total cost. - CHE incidence was 40% and ranged between 58% and 20% among the poorest and richest income quintiles.	- Private facility diagnosis, extra- pulmonary TB, hospitalized patients, being poorest, and TB/HIV co-infection was very likely to have TB- related CHE. - Every additional visit for TB diagnosis increases the odds of experiencing CHE by 2.4 times. - Households with a health insurance scheme have protection from CHE.
**Lelisa, 2020** (10)	Extended cost-effectiveness analysis modeling study.	TB, DS-TB, and MDR-TB patients.	NA	NA	- Active case finding could reduce TB-related deaths and catastrophic costs by 27% and 32%. - Enhancing DOTs for DS-TB would avert 25% of deaths and 15% of catastrophic costs. -Improvements in MDR- TB care would avert up to 1% and 6% of all deaths and catastrophic costs, respectively, over 2018- 35.	NA
**Mengiste, 2010** (24)	Cross-sectional survey.	New pulmonary tuberculosis patients ≥ 15 years old.	10 districts of Tigray region.	− 924 newly diagnosed PTB patients (537 smear-positive and 387 smear-negative PTB). -Median age was 34 years.	- The total median cost incurred from the first consultation to diagnosis was $27 per patient (mean = $59). - The median costs per patient incurred by a patient, escort, and the public health system were $16 (mean = $29), $3 (mean = $23), and $3 (mean = $7) respectively. - The indirect and direct costs comprised 61% and 39% of the total cost spent to diagnose TB patients.	The total cost per patient diagnosed was higher for women, rural residents; those who received government food for work support, patients with smear-negative pulmonary tuberculosis, and patients who were not screened for TB in at least one district diagnostic center.
**Tsegahun, 2022** (15)	Open label RCT.	Adults aged 18 years or older with new or previously treated, bacteriologically confirmed, and drug-sensitive pulmonary TB.	10 health care facilities in Ethiopia.	-109 participants. -Age- mean (SD) 33.1 (11.1) years.	-Median postdiagnosis cost was US $1.53. − 42 (38.5%) faced catastrophic costs due to TB treatment.	- Occupation, number of cohabitants, and smoking.
**Van, 2016** (23)	Cross-sectional survey.	TB and MDR-TB patients.	St. Peters and ALERT in Addis Ababa and University of Gondar Hospital in Gondar.	− 169 MDR-TB patients and 25 other TB patients. - Age- NA	**-**Total pre-diagnosis and treatment costs 260 USD. -Total pre-diagnosis costs of TB median (IQR) 14 (6–129). - Total pre-diagnosis costs for MDR-TB median (IQR) 68 (35–191). -Total pre-diagnosis and treatment costs for MDR-TB 1838 US$. **-** Percentage of patients reporting income loss due to TB was 92% and 79% due to MDR-TB.	NA
**Vassall, 2010** (21)	NA	Patients aged ⩾15 years who starting TB or HIV treatment.	Addis Ababa, Hosanna, and Jimma (urban, rural, and peri- urban).	-93 patients starting TB or HIV treatment. - Age- NA	- Pre-treatment costs were 35% of annual household income for TB patients (with no HIV) and 33% for those with TB and HIV. - Total mean pre-diagnosis cost for TB patients was US$ 129 and 170 for TB-HIV co-infection	NA

**ALERT**: All Africa Leprosy, Tuberculosis, and Rehabilitation Training Centre; **CHE**: Catastrophic Health Expenditure; **DOTs**: Direct Observed Therapy; **DS-TB**: Drug drug-susceptible TB; **HIV**: Human Immunodeficiency Virus; **IQR**: Interquartile range; **MDR-TB**: Multidrug resistance TB; **NA**: Not available; **PTB**: Pulmonary TB; **RCT**: Randomized Control Trial; **SD**: Standard Deviation; **TB**: Tuberculosis; **US**: United States

**Table 2:** Methodological quality assessment for cross-sectional studies

Included Studies	Selection				Comparability	Outcome		Score
	Representativeness of the sample	Sample size justified	Non-response	Ascertainment of exposure (max**)	Confounding controlled (max**)	Outcome assessment (max**)	Statistics	Total
**Belete, 2010**(27)	*	*	*	**	**	*	*	9/10
**Collins, 2018** (18)	**-**	**-**	*	**	**-**	*	**-**	4/10
**Lelisa, 2020** (25)	*	*	*	**	**	**	*	10/10
**Mengiste, 2010** (24)	*	*	*	**	**-**	*	*	7/10
**Van, 2016** (23)	*	*	*	**	**-**	*	**-**	6/10

### TB-related catastrophic cost

**Fig. 2:**
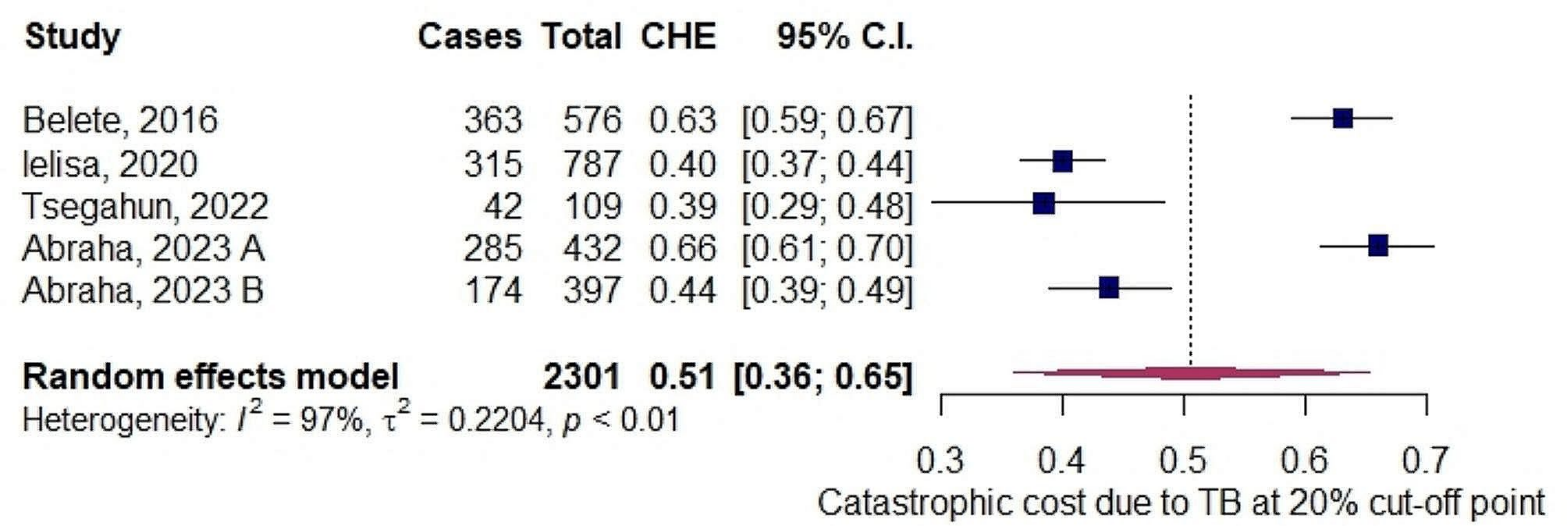
Forest plot of pooled prevalence of catastrophic cost due to bacteriologically confirmed drug-sensitive pulmonary TB illness in Ethiopia

Our meta-analysis showed that the proportion of patients facing catastrophic costs due to bacteriologically confirmed drug-sensitive pulmonary TB at a cut-off point of 20% was 51% (5 studies, 2301 participants, 95% CI, 36-65%, I^2^ = 97%)( Fig. 2). However, at the 10% threshold of total expenses, 48% (353 households) of TB households experienced catastrophic total costs [25]. From the results it is evident that private facility diagnosis, occupation, extra-pulmonary TB, smoking, number of cohabitants, hospitalized patients, and being poorest were very likely to have TB-related catastrophic health expenditure (CHE) [21]. Moreover, the incidence was much higher for those with TB/HIV co-infection (48%), DR-TB (62%), and (94%) hospitalized TB patients [25]. The odds of experiencing CHE increases by 2.4 times in every additional visit for TB diagnosis [25]. Similarly, on average, 33-76% of annual household income has been lost due to illness and TB treatment [21, 23], and around 72% due to MDR-TB illness [23]. On the contrary, active case finding and households with a health insurance system could reduce TB-related deaths and catastrophic costs [10, 15, 25].

### Pre-diagnosis cost

Up until TB diagnosis, patients faced median (IQR) costs of US$97.6 (56.4-184.3) [4] and US$14 (6-129) for drug sensitive TB, while MDR-TB incurred costs of US$75 (40–191) [23]. The overall mean pre-diagnosis cost for drug sensitiveTB patients consistently ranged from US$59 to US$129 [21, 24]. For TB/HIV co-infected patients, the cost was slightly higher, ranging from US$170 to US$188 [21, 25]. Direct costs were notably significant [21] and comprising 25.6-39% of the total pre-diagnosis cost [4, 24]. Additionally, pre-diagnosis medical, non-medical, and indirect costs constituted 11.1%, 14.5% [4], and 61-74.4%, respectively, of the total pre-diagnosis cost [4, 8, 20, 24].

The pre-diagnosis cost is strongly positively correlated with patient and provider delays, total delays, and the number of healthcare facilities visited until diagnosis [4]. Patients not screened at the first diagnostic centers had higher patient and escort costs per patient [24], and also, the pre-diagnosis cost for those using alternate care providers or misdiagnosed in two or more public health facilities was 3.6 times higher [24]. Mean pre-diagnosis cost increased by 0.5% for each patient and provider delay day. Clinically diagnosed patients incurred 11% higher mean pre-diagnosis costs than those diagnosed bacteriologically. Pre-diagnosis costs were 18% higher for patients diagnosed at private healthcare facilities compared to public ones [4], varying significantly across types of TB, first visited healthcare facility, facility of diagnosis, and mode of diagnosis [4]. Among the factors contributing to high mean pre-diagnosis costs, food expenditure, and supplements during treatment were the highest for both TB and MDR-TB patients [23].

### Post-diagnosis cost

**Fig. 3:**
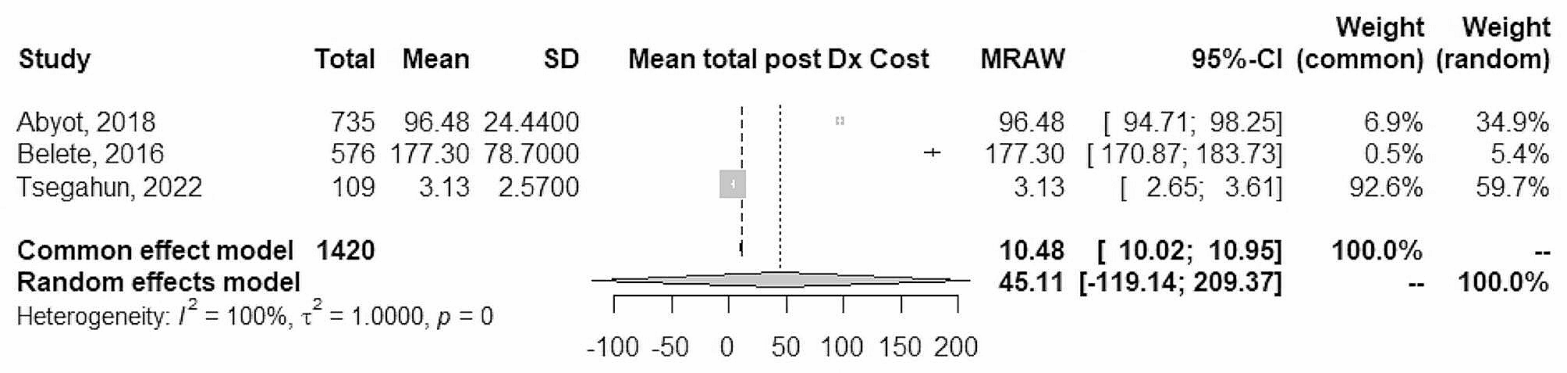
Forest plot of total post Dx cost

Data from 3 studies were combined using a random effects model with the inverse method of variance, estimating a mean total post-diagnosis cost of 45.1 US$ (95% CI between − 119.1 and 209.37). There is high heterogeneity among the combined studies (Tau ^2^ = 1; p-value = 0; I^2^ = 100%)(Fig. 3).

However, out of the total post-diagnosis cost, direct and indirect costs constituted 35.9% and 64%, respectively [4] and most of the patients spent a lot of money during the intensive phase (first two months of TB treatment), but declined drastically over time [16, 18, 21]. During the continuation phase, when symptoms are less severe, indirect costs decrease [21]. Compared to smear-positive patients, the mean unit patient, escort, and public health system costs for smear-negative PTB patients were much higher [27]. However, it was untoward that some patients lost up to 48% of annual household income due to TB treatment [21].

Compared to those being urban residents, the mean post-diagnosis cost increased by 48% for those patients who were rural residents, having a travel time beyond 1 h to the treatment center increased. The mean post-diagnosis cost was higher by two folds for those patients hospitalized for anti-TB treatment. Whereas, it was lower by 18% for those patients who had been following their anti-TB treatment at hospitals compared to those who received it at health centers [4]. This was due to having full-time staff that fully provides TB patient care at hospitals. Besides, in health centers healthcare providers are given multiple responsibilities, other than TB DOTs program which increase patient waiting time and costs, and these health centers are located in a rural place where access to transportation is very limited within villages compared to hospitals in urban areas [4].

Similar to pre-diagnosis cost, the mean post-diagnosis cost increased by 0.3% and 0.2%, respectively due to patient and provider delays independently [4]. In addition, there was a significant association between total post-diagnosis cost and the patient’s household income, residence, need for additional food, and primary income [21, 27].

On the contrary, a significant reduction in the total post-diagnosis cost (US$24.4 Vs US$8.4) has been observed by switching the TB DOTs scheme from a healthcare facility to a community TB DOTs scheme (16). Furthermore, for those patients who have completed their primary and higher education and were treated at a hospital the mean post-diagnosis cost was much lower [4].

### Total cost

**Fig. 4:**
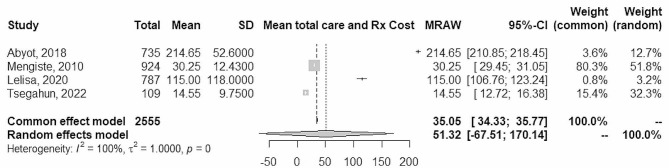
Forest plot of total care seeking and treatment

Data from 4 studies were combined using a random effects model with the inverse method of variance, estimating a mean total cost incurred by the patients due to TB care seeking and treatment of 51.3 US$ (95% CI between − 67.5and 170.14). There is high heterogeneity among the combined studies (Tau ^2^ = 1; p-value = 0; I^2^ = 100%) (Fig. 4).

**Fig. 5:**
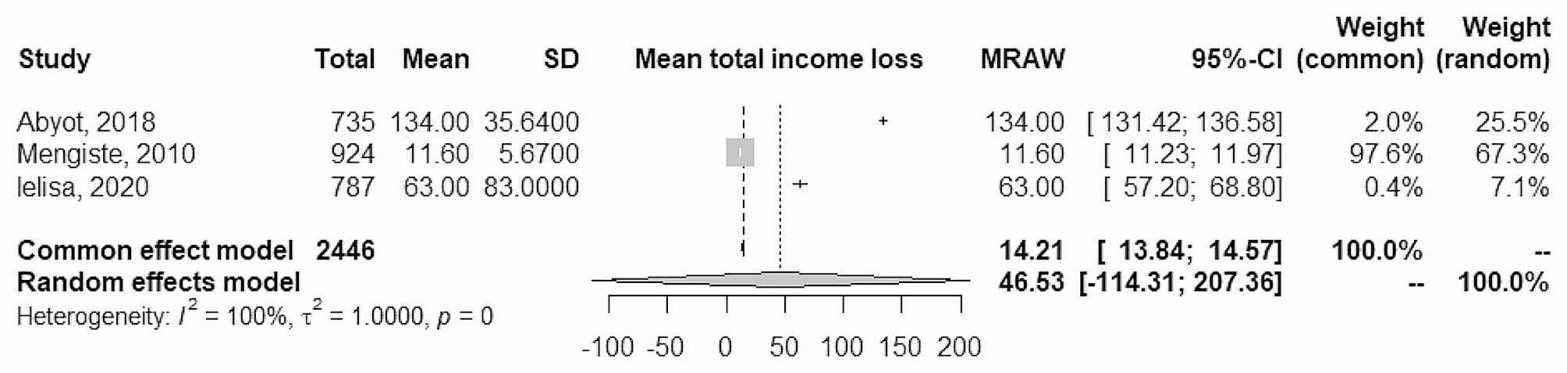
Forest plot of total mean income loss

However, the total cost of care significantly varies by type of TB, it was US$104 (US$107) for pulmonary, US$140 US($138) for extra-pulmonary, and US$446 (US$732) for DR-TB [25]. Consistently, compared with pre-treatment level, TB patients’ mean productivity and income reduced by 37% and 10%, respectively, whereas mean household expenditure increased by 33% and working hours reduced by 78% due to TB illness [27]. Data from 3 studies were combined using a random effects model with the inverse method of variance, estimating a mean total income loss was 46.5 US$ (95% CI between − 114.3 and 207.4). There is high heterogeneity among the combined studies (Tau ^2^ = 1; p-value = 0; I^2^ = 100%) (Fig. 5).

Among the out-of-pocket cost (OOP), food supplements for nutrition support and hospital-related direct cost covers 37% and 33.6%, respectively [27]. Although out of the total pre- and post-diagnosis cost, the direct cost ranges from 29 to 46% [4, 25]. Similarly, 49.7% and 44.6% of the total medical costs had independently correspond to drugs other than anti-TB and diagnostic tests (laboratory or imaging tests) and the total cost represents more than 10% of their estimated household annual income for 471/569 (82.8%) of the cases [4].

The mean total patient cost of TB care independently associated with rural residence, travel time to treatment center beyond 1 h, action taken before HCF visit, hospitalization for anti-TB treatment, number of visited HCF, and patient and provider delays. Consistently, the mean total cost incurred by patients who are rural residents is about 24% higher than that by urban residents and every patient and provider delay day expect to increase the mean to total patient cost by 0.3%. Furthermore, those patients who visited a healthcare facility before initiating HCF visits incurred 17% higher mean total cost compared to those who did not visit. Likewise, the total mean patient cost was higher by 97% for those patients who were hospitalized during anti-TB treatment compared to those not hospitalized [4].

Even though initial investment for implementation is required for the community-based DOT program, training, and supervision, the total cost for each successfully treated smear-positive patient was higher in health facilities (US$161.9) compared with the community-based approach (US$60.7) [16]. The total cost, patient cost, and caregiver costs of community-based treatment were lower than health facility DOT by 62.6%, 63.9%, and 88.2%, respectively [16]. However, involving health extension workers (HEWs) added a total cost of US$8.80 to the health service per patient treated in the health posts. Involving HEWs in TB treatment is a cost-effective treatment alternative for the health service, the patients, and the caregivers [16].

### Cost for MDR-TB

In terms of the cost related to MDR-TB infection, the cost was greater with the long than the short regimen [17]. The total median (IQR) cost during pre-diagnosis, intensive, and continuous phase were US$83 (40–206), US$661 (269–968), and US$634 (458–1048), respectively [18]. Consistently, the total direct and indirect costs were US$1378 [18] and direct costs per participant were US$ 575.4 for the long regimen and US$ 337.3 for the short regimen [17]. For those patients who were treated with the longer regimen the mean cost of a serious adverse event was higher than the short regimen [17]. The short MDR tuberculosis treatment regimen corresponded with a significant reduction in health-system costs and a lower financial burden for participants [17]. Also, due to the high cost of treatment 73% of MDR-TB patients in Ethiopia received assistance [23]. But, the percentage of patients reporting income loss due to MDR- TB was 92%, and 79% due to TB [23]. Furthermore, in the capital Addis Ababa, the costs of treatment of adverse events, patient transport, patient accommodation, and supplementary food were much higher [18].

### Coping costs

The impact of tuberculosis infection on patient employment, and overall patient and family income was significant. Due to TB/MDR-TB illness, some patients sold property, lost their jobs, borrowed money, lost their household income, used their savings to cope with the costs, and took out loans [18, 23, 25, 27].

### Mitigation plans

To reduce TB-related death and catastrophic cost, enhancing early detection strategy would be very critical [10]. According to a recent extended cost-effectiveness analysis modeling study report, between 2018 and 35, in Ethiopia, improving DOTs would prevent 25% of deaths and 15% of catastrophic costs; and enhancements in MDR- TB care would also prevent up to 1% and 6% of all deaths and catastrophic costs, respectively [10]. To improve healthcare services for TB patients, we need to make sure that policy of free care for all MDR-TB services is fully implemented, bring services closer to patients, detect and treat MDR-TB cases earlier, and raise the awareness of health workers [23]. Furthermore, to the enhance overall income of the patients, implementing social protection strategies including direct (transport, food support) costs in social support schemes provided through TB services and indirect (sick leave allowance) costs in social protection schemes like utilization of social health insurance, improving employment protection, reduction of stigma, acceptance of outpatient treatment, increasing re-socialization and employment possibilities, and consistency across social assistance programs would also be necessary [23].

## Discussion

Tuberculosis (TB) imposes substantial and often catastrophic costs on patients. Our meta-analysis reveals that, with a 20% threshold, 51% of patients faced catastrophic costs (5 studies, 2301 participants, 95% CI, 39-62%). Out-of-pocket payments proved to be overwhelmingly burdensome for most TB patients. Factors such as private facility diagnosis, occupation, extra-pulmonary TB, smoking, cohabitant numbers, hospitalization, and socioeconomic status were strongly associated with TB-related catastrophic health expenditure (CHE). It was higher for TB/HIV co-infection, drug-resistant TB (DR-TB), and hospitalized TB patients. The pre- and post-diagnosis costs, covering medical, non-medical, and indirect costs, constitute a quarter of the overall pre- and post-diagnosis expenses. Importantly, these costs correlate positively with patient, provider, and total delays, as well as the number of healthcare facilities visited until diagnosis. Furthermore, the total post-diagnosis cost of TB is significantly influenced by factors such as the patient’s household income, residence, dietary needs, hospitalization, the intensive phase of treatment, and primary income sources.

In a WHO survey spanning 27 countries burdened with tuberculosis (TB), the combined average for all 27 nations, adjusted for each country’s reported cases, was 48% (95% CI: 36–61% [5]. Among the 23 countries that supplied detailed data, the percentage of individuals facing catastrophic total costs was notably elevated for those with drug-resistant TB (DR-TB), with a consolidated average of 82% (95% CI: 75–90%) [5]. Similarly, a recent meta-analysis and systematic review demonstrated that, at a 20% threshold, 43% of patients with drug-sensitive TB, 80% with MDR-TB, and 81% with TB/HIV co-infection experienced catastrophic costs [6, 28]. Our findings align with previous studies conducted in low-income settings such as Nigeria [29] and Uganda [30]. However, the observed rates were comparatively higher than those reported in Kenya [31] and and lower than those in Benin [32].The catastrophic expenses were higher among patients who have waited for more than four weeks following the onset of symptoms to start treatment and severe symptoms, prolonged hospitalization, more expensive non-TB medication, or even more frequent visits to the facilities may also explain why delayed treatment initiation was related to catastrophic expenses [29]. On the contrary, emphasizing relevant public social protection programs, health care financing, and health and social protection investments may potentially be a solution in mitigating the negative impact of TB in the pursuit of TB elimination.

A substantial financial costs incurred across pathways to TB care by patients and their families with a significant burden of TB and poverty. Affordable health services are needed to enable access, reduce delays, and compensate for direct and indirect costs [9]. This study revealed that the mean of income loss (from three studies) were US$45 and estimated mean of total costs (from four studies) were US$51. Income loss due to TB illness contributes significantly to the total costs associated with tuberculosis. The financial burden imposed by TB goes beyond direct medical expenses and includes indirect costs such as income loss. Individuals diagnosed with TB often face challenges in maintaining their regular work or employment during the period of illness, leading to income reduction or loss [9, 33]. The total cost incurred across the care-seeking and treatment pathways is also significantly correlated with both patient and provider delays. The increased risks of severe manifestation may also increase pre-diagnosis cost with patient delay [34, 35] that lead to hospitalization and companionship during care seeking and treatment. Besides, patient delay is associated with informal care including self-treatment and traditional care [36] that pose costs to patients. One study from Pakistan has shown that the cost during the pre-diagnostic phase and the indirect cost contribute 49% and 42% of the total household out-of-pocket payment for TB care [37]. Previous reports have also showed the higher cost of the post-diagnosis DOT strategy [9, 38–41]. The cost incurred by TB patients also depends on the kind of pace where these patients came from. For instance, TB patients living in central Ethiopia are facing multiple challenges, and the cost was even quite higher than the estimated for PTB patients in the Southern region [16] and the $53 estimated for PTB patients in 10 district areas of Tigray region [24] and Ethiopia, though it was by far lower than the $847 estimated for low-income countries [9].

Despite the cost incurred by the DOT strategy, transportation and food cost related to long-distance traveling to the health facilities, the cost of diagnosis in the private sector, and hospitalization had also a significant impact on the out-of-pocket cost by the patients [27, 42, 43]. These kinds of challenges could enforce the patients to stop their treatment even if they understood the consequences, which might lead them to develop multi-drug resistance tuberculosis infection and XDR-TB. However, rearranging the DOT schedule to weekly or monthly intensive phase, ensuring the provision of free-of-charge TB diagnosis and treatment in private facilities, community-based treatment of TB patients through health extension workers, workplace-based treatment, bringing services closer to patients, transportation reimbursement schemes and food assistance, and evidence-based cost-effective diagnostic and treatment routines might have a possibility to reduce the pre and post diagnosis out of pocket payment and retain TB patients in care [9, 16, 27, 44, 45].

When a substantial number of people facing TB incur catastrophic costs, it triggers a cascade of social and economic challenges. These costs plunge individuals into poverty, hinder timely healthcare seeking, intensify health issues, and elevate TB transmission risks. Productivity dwindles, stigma rises, and existing health disparities widen [46–49]. The economic burden impedes TB control efforts, potentially leading to delayed diagnosis and incomplete treatment [46–49]. Mental health struggles add another layer to the overall impact. Addressing these issues necessitates innovative strategies encompassing financial support, community education, and policies to alleviate the economic strain on TB-affected individuals and communities.

This study has some limitations. Since we have only included five studies in the meta-analysis of catastrophic cost, the pooled estimate has substantial heterogeneity. Given the heterogeneity observed in our meta-analysis results, we do not assert that this study can replace a national cost survey. Nevertheless, it can significantly contribute to presenting a comprehensive overview of catastrophic costs and their associated factors for policymakers, healthcare professionals, donors, and organizations dedicated to TB in Ethiopia. Besides, most of the studies were cross-sectional which might also reduced the quality of evidence generated from this review. The utilization of the 20% threshold set by the WHO to identify catastrophic costs might underestimate the actual burden in Ethiopia. This is because contextual factors like the prevailing level of poverty and existing social protection interventions could influence the extent of catastrophic costs. Besides, half of the studies used the Stop-TB partnership tool to estimate the cost, but the rest of the studies used other tools which might make their results heterogenious. Furthermore, we couldn’t able to pool the pre-diagnoisis and other types of cost because the data reported by majority of the studies were skewed.

## Conclusion

More than half of TB patients were suffering from out-of-pocket payments with catastrophic consequences in Ethiopia and most of the patients spend a lot of money during the pre-diagnosis period and intensive phase, which in turn were hampering the efforts to end TB. The financial burden of MDR-TB is alarming, although all TB patients experienced a substantial socioeconomic impact of the disease. Promoting active case finding and home-based DOT programs, patient-centered digital medication event reminder and monitor-observed therapy, promoting early care-seeking behavior, promoting patient-centered care with comprehensive health insurance coverage, promoting patient-centered digital health technologies, ensuring providing diagnostics free of charge, decentralizing efficient diagnosis, providing social support at the start of treatment, nutrition support, bringing services closer to the patient, and introducing reimbursement system for direct costs can help minimize financial burden to the patient and could have the potential to overcome challenges which might hamper the End TB strategy.

## Data Availability

Data will be available upon request from the corresponding authors.

## Abbreviations

ALERT: All Africa Leprosy, Tuberculosis, and Rehabilitation Training Centre
CENTRAL: Cochrane Center for Clinical Trial database
CHE: Catastrophic Health Expenditure
CI: Confidence interval
DOTs: Direct Observed Therapy
DR- TB: Drug resistance TB, DS-TB:Drug-susceptible TB
HCF: Health care facilities
HEW: Health extension workers
HIV: Human Immunodeficiency Virus
IQR: Interquartile range
MDR-TB: Multidrug resistance TB
MeSH: Medical Subject Headline
NA: Not available
OOP: Out of pocket cost
PICOS: Patient, Intervention, Comparison, Outcome, and Study
PRISMA: Preferred Reporting for Systematic Review and Meta-analysis
PROSPERO: Prospective Register of Systematic Reviews
PTB: Pulmonary TB
RCT: Randomized Control Trial
RR: Risk ratio
SD: Standard Deviation
TB: Tuberculosis
US: United States
WHO: World Health Organization
XDR-TB: Extensively drug-resistant TB
